# Pilot Multimodal Imaging Assessment of Neuropathological Iron Deposition and Molecular PET in Frontotemporal Lobar Degeneration

**DOI:** 10.3390/brainsci16101013

**Published:** 2026-09-24

**Authors:** Rodolfo G. Gatto, Hossam Youssef, Gokhan Uruk, Arenn F. Carlos, R. Ross Reichard, Val J. Lowe, Jennifer L. Whitwell, Keith A. Josephs

**Affiliations:** 1 Department of Neurology, Mayo Clinic, College of Medicine and Science, 200 1st Street S.W., Rochester, MN 55905, USA; youssef.hossam@mayo.edu (H.Y.); uruk-contreras.gokhan@mayo.edu (G.U.); josephs.keith@mayo.edu (K.A.J.); 2 Department of Laboratory Medicine and Pathology, Mayo Clinic, Rochester, MN 55905, USA; reichard.robert@mayo.edu; 3 Department of Radiology, Mayo Clinic, Rochester, MN 55905, USA; vlowe@mayo.edu (V.J.L.); whitwell.jennifer@mayo.edu (J.L.W.)

**Keywords:** frontotemporal lobar degeneration, Alzheimer’s disease, iron aggregation, positron emission tomography, neuropathology, immunofluorescence, confocal microscopy, diffusion MRI

## Abstract

**Highlights:**

**What are the main findings?**

- Iron accumulation was associated with tau pathology in FTLD.
- Ferritin frequently colocalized with pathological tau aggregates.

**What are the implications of the main findings?**

- Altered iron homeostasis may contribute to FTLD pathology.
- Larger studies are needed to validate these preliminary findings.

**Abstract:**

Background/Objectives: The role of iron in Alzheimer’s disease (AD) and frontotemporal lobar degeneration (FTLD) has been debated. However, the effects of iron on protein burden in these diseases remain poorly elucidated. We hypothesized that there is an association between iron deposition and tau and beta-amyloid deposition in deep grey matter (GM) regions in FTLD. Methods: Neuropathological samples were collected from two control subjects and 10 disease subjects. This included 12 subjects, with several pathological subgroups represented by only one or two cases. Of the 10 disease subjects, one had low-level AD neuropathology (L-AD), and one had high-level AD neuropathology (H-AD), whereas the remaining eight subjects had FTLD-tau pathology, including progressive supranuclear palsy (PSP), corticobasal degeneration (CBD), globular glial tauopathy (GGT), and Pick’s disease (PiD). Neuropathological samples were stained for iron with Prussian blue (PB) and ferritin, tau, and beta-amyloid in the basal ganglia (BG). All 12 subjects had also undergone diffusion MRI, amyloid-PET (PiB-PET), and Tau-PET (flortaucipir) before death. Diffusion tensor imaging mean diffusivity (MD) from BG was examined in each subject. Spearman’s rank correlations between imaging and histology were calculated. Results: Increased BG iron was observed in PSP subjects. Prussian blue and ferritin measures were associated with higher tau- and β-amyloid pathological burden. Tau-PET uptake correlated with iron in the BG. Conclusions: The results of this exploratory study suggest associations between iron burden and tau- and β-amyloid-related measures within basal ganglia regions that may help to explain increased PET signals in AD and FTLD-Tau. Our findings should be considered to be hypothesis-generating and require validation. These preliminary findings require validation in larger cohorts.

## 1. Introduction

Iron is a critical element for normal cellular function and is present in neurons and glial cells [1]. Proper iron homeostasis is required for the binding of diverse proteins involved with maintaining adequate levels of brain oxygenation and metabolism [2]. Moreover, local iron redistribution is related to regional brain tissue requirements, ensuring an adequate supply of oxygen in regional brain tissue with high activity/demand [3]. Therefore, iron dysregulation could be an important contributor to the development of neurodegenerative pathologies [4,5,6,7].

In normal aging, increased levels of brain iron have been related to atrophy, disrupted neural activity, and motor and cognitive deficits, pointing to the harmful effects of higher iron concentration on brain integrity and motor and cognitive performance. An imbalance of iron metabolism has been previously associated with aging and cognitive deficits [8,9,10], whereas the disruption of iron homeostasis has been linked to the onset and progression of a variety of psychosocial disorders. In particular, the accumulation of iron in the basal ganglia (BG) has been related to impairments in spatial, aversive, and recognition memory [11]. Reports have demonstrated a correlation between increased deep grey matter (GM) iron deposition and poor memory performance, as well as between iron deposited in the putamen (Pu) and the globus pallidus (GP) and general cognition [10]. Additional studies have reported that iron accumulation is one of the key factors linked to cognition in older patients who are non-demented [12]. As such, the lack of iron homeostasis is a plausible mechanism for cognitive impairment and neurodegeneration, particularly in the aging population [13]. As such, the accumulation of iron has often been observed in patients with dementias including those with Alzheimer’s disease (AD) [14,15,16,17] and frontotemporal lobar degeneration (FTLD) [18,19,20].

In the context of AD, tau forms intracellular insoluble aggregates known as neurofibrillary tangles (NFTs), a process that appears in many cases to be preceded by hyperphosphorylation of tau monomer [21]. Intrinsically, tau phosphorylation processes are balanced by tau kinase and phosphatase activities [22]. Disruption of this balance has been suggested to be the basis of abnormal tau phosphorylation leading to tau aggregation. Previous studies have reported that iron can induce aggregation of soluble paired helical filament tau [23], and that iron can regulate tau phosphorylation by inducing the activity of multiple kinases that promote tau hyperphosphorylation, potentially impacting protein phosphatase 2A activity [24]. Iron is also reported to induce the aggregation of hyperphosphorylated tau, through direct interaction via a recognized iron binding site in the tau protein, facilitating the intracellular formation of NFTs [25]. Ultimately, beta-amyloid and hyperphosphorylated paired helical filament tau (PHF-1) can lead to iron deposition in the brain [26] and therefore diverse catalytic neurodegenerative processes [27]. There is mounting evidence linking an increase in brain iron content and the formation of phosphorylated PHF-1 in patients with different types of dementias [12]. Regarding FTLD, there has been a reported association between tau deposits in FTLD-tau, and iron accumulation in cortical and subcortical regions related to specific genetic mutations [18,28]. As such, imaging-pathological studies of iron deposition in FTLD-tau have found a prominent irregular hypointense signal in the deeper cortex, distinct laminar patterns of novel iron-laden glial pathology, and iron-positive ameboid and hypertrophic glia in deeper GM and adjacent WM regions [29].

Structural MRI-based methods are commonly used to interrogate microstructural patterns across subjects affected by neurological diseases [30]. MRI studies have found that iron accumulation may lead to further deposition of beta-amyloid and the formation of NFTs in AD [31,32]. Among different diffusion MRI (dMRI) techniques, diffusion tensor imaging (DTI) has been described as useful for evaluating brain microstructure affected by tau deposition [33,34]. In addition, brain deposits of beta-amyloid and tau can be measured by positron emission tomography (PET) [35,36,37,38].

In this study, we proposed that the combination of structural MRI and biochemical methods (PET) supported by neuropathological findings can be particularly useful for the comprehensive study of the interplay between iron and other molecular biomarkers in the context of FTLD-tau. To the best of our knowledge, this is the first study to compare neuropathological iron depositions with ante-mortem molecular PET in FTLD-tau patients. We hypothesize that there will be an association between iron deposition and tau and beta-amyloid in the BG in FTLD-tau patients.

## 2. Materials and Methods

### 2.1. Clinical Cases

Brain tissue from the neuropathological database at the Mayo Clinic, Rochester, Minnesota, was collected from twelve participants who had been enrolled in an NIH-funded study by the Neurodegenerative Research Group (NRG; PIs: Josephs & Whitwell), had died with brain autopsy examination between 1 January 2017, and 31 July 2022, and had completed imaging studies including antemortem MRI scan, flortaucipir PET scan (Tau-PET), and Pittsburgh compound B beta-amyloid PET (PiB-PET) scan. The study was approved by the Mayo Clinic IRB, and all participants or proxies had consented to research; proxies provided consent for patients when needed. The study followed the ethical standards of the Committee on Human Experimentation at Mayo Clinic by the Helsinki Declaration of 1975. While part of the cohort has been described previously [39], the present study focuses on a different research question and presents novel imaging and statistical analyses that were not included in the earlier report. Throughout the manuscript, Alzheimer-related pathology is described using the abbreviations low-likelihood AD (L-AD) and high-likelihood AD (H-AD) for consistency.

### 2.2. Neuropathological Studies

All cases had a standardized neuropathologic evaluation following accepted, published methodologies [40]. Basal ganglia subregions, including the globus pallidum internus (GPi), globus pallidum externus (GPe), and putamen, were chosen to determine the prevalence of iron deposition across all twelve subjects. Samples were examined following standard cortical sampling, according to the Consortium to Establish a Registry for Alzheimer’s Disease (CERAD) [41,42].

For each brain region, iron staining was performed with Gomori’s Prussian blue (PB). This iron stain detects iron ions in loosely bound protein complexes, such as hemosiderin. Iron that is strongly bound, such as iron in hemoglobin, will not react [43,44]. Tissue sections were treated with an acidic solution of potassium ferrocyanide. Thus, the iron present in the tissue sections will react to form an insoluble bright blue pigment called PB [45]. Nuclear Fast Red (NFR) was used as a counterstaining method [46]. Liver tissue was processed using the Ventana BenchMark automated stainer (Ventana Medical Systems, Inc., Tucson, AZ, USA) for quality control purposes. Details of this staining can be retrieved elsewhere [47].

To assess tau burden, DAB (3,3′-diaminobenzidine) staining was applied with (1) anti-phosphor-PHF-tau pSer202 + Thr205 antibody, clone AT8, mouse-host monoclonal, (Thermo Fisher Scientific, Waltham, MA, USA, MN1020, 1:100) [48]; (2) anti-beta-amyloid was detected by a monoclonal mouse clone 6F/3D antibody (Dako, Agilent Technologies, Glostrup, Denmark, M0872, 1:50) [49]. Histopathological imaging was performed using a scanning microscope (Grundium Ocus^®^40 Digital pathology microscope scanner, Tampere, Finland). Five (5) different, randomly selected regions of interest (ROI) in each histology section were photographed and averaged to obtain a final score per sample. This was followed by quantitative histological analysis using threshold and masking methods with ImageJ software, version 1.54r (National Institutes of Health, Bethesda, MD, USA) [50,51] with standard methods described elsewhere [49,52]. Because cases with matched ante-mortem MRI, Tau-PET, PiB-PET, and postmortem neuropathology are exceptionally rare, individual subject data were retained whenever possible to preserve biological variability and disease-specific findings. The four PSP and two GGT cases were pooled only for selected descriptive visualizations and summary tables to facilitate interpretation and were not used for formal group-level inference. Given the heterogeneity of FTLD-tau syndromes, individual results are presented throughout the manuscript whenever feasible, with pooled summaries provided solely as complementary descriptive representations. Histological image quantification and colocalization analyses were performed without knowledge of PET imaging results; although neuropathological diagnoses were available during tissue evaluation, they were not considered during quantitative image analysis.

### 2.3. Immunofluorescence and Confocal Studies

Consecutive slides were sectioned from the same block used in neuropathology staining for comparative analysis. Following general deparaffinization of all samples, permeabilization and retrieval with citrate and EDTA was done at room temperature for 20 min. Samples were then stained with: (1) an anti-phosphorylated tau AT8 mouse host monoclonal antibody (Thermo Fisher Scientific, catalog #MN1020, 1:250) to detect phosphorylated tau [48], (2) anti 4RTau (RD4) monoclonal antibody, mouse host (Millipore, Billerica, MA, USA, Clone 1E1/A6, catalog #05-804, 1:50) that recognizes truncated-tau [53], and (3) anti-ferritin light chain polyclonal antibody rabbit host (Abcam, Cambridge, UK, catalog #sb69090; 1:500) [54]. Secondary antibodies, goat anti-mouse 488 nm (green channel) or anti-rabbit Alexa 594 nm (red channel) at 1:200, were applied after incubation. Each specimen was incubated with TrueBlack (Biotium, Fremont, CA, USA, catalog #23007) for 30 min to reduce lipofuscin and other autofluorescence pigments. Confocal microscopy was performed with an LSM 780 microscope from Zeiss (Carl Zeiss Microscopy GmbH, Jena, Germany). Images were acquired at a resolution of 1024 pixels (0.35 × 0.35 microns). For quantitative colocalization analysis, four representative microscopic fields were acquired from each GPi, GPe, and putamen section using identical microscope acquisition settings. Image preprocessing and colocalization analyses were performed in ImageJ. Background signal was minimized through standard image preprocessing procedures, including removal of nonspecific background fluorescence before analysis. Thresholds for each fluorescence channel were applied using identical predefined settings across all cases and regions to ensure consistency of measurements. Pearson’s colocalization coefficient (PCC) was calculated between the AT8 and ferritin fluorescence channels for each field. The PCC values reported represent the mean PCC obtained from the four analyzed fields within each anatomical region for each individual subject. Because several pathological categories were represented by only one or two subjects, these values are presented as individual case-level regional measurements and should not be interpreted as disease-group estimates. Quantitative image analysis was performed without knowledge of the final quantitative results at the time of image acquisition and processing. Imaging preprocessing analysis was done with the ImageJ software as described elsewhere [49]. For quantitative analyses, image acquisition and region selection were performed using identical microscope settings and predefined anatomical landmarks. Fields were selected independently of staining intensity and analyzed using consistent thresholding parameters across all cases.

### 2.4. Diffusion Tensor Imaging Studies

All cases and controls underwent a standardized MRI protocol on a 3T Prisma scanner (Siemens Healthineers, Erlangen, Germany). The imaging protocol included a 3D Magnetization Prepared Rapid Acquisition Gradient-Echo (MPRAGE) sequence (TR/TE/T1 = 2300/3/900 ms; 26 cm FOV, slice thickness = 1.2 mm, in-plane resolution = 1 mm) as well as a diffusion MRI (dMRI) sequence with a spin-echo single shot Echo Planar Imaging (EPI) sequence. TE = 71 ms, and TR = 3400 ms. Each dMRI scan included a multi-shell diffusion scheme with diffusion-weighted b values: b = 500, 1000, and 2000 s/mm^2^. The number of diffusion sampling directions was 6, 48, and 60, respectively. The in-plane resolution was 1.36 mm scanned with 2.0 mm isotropic voxels, with a slice thickness of 2 mm. Images were corrected for head motion, Eddy current, and susceptibility artifact distortion using standard algorithms extensively described elsewhere [55]. Additionally, diffusion data was checked by an automatic quality control routine to ensure its accuracy [56]. A diffusion tensor imaging (DTI) model was estimated and subsequently used to calculate mean diffusivity (MD) employing open-source software (DSI Studio: https://dsi-studio.labsolver.org), using the 2024.05.22 “Chen” release version and accessed on 18 March 2026 following standard methods [57]. Regional diffusion metrics were calculated for the Pu, GPi, and GPe subregions using the FreeSurfer Desikan–Killiany–Tourville (DKT) subcortical anatomical labeling atlas [58]. On occasion, due to gross anatomical degeneration and deformations, manual ROI corrections and realignments were performed by experienced users familiar with neuroanatomical landmarks.

### 2.5. PET Imaging Studies

All PET scans were acquired using a PET/CT scanner (Siemens Healthineers, Erlangen, Germany). Each case was injected with ~370 MBq (range 333–407 MBq) of 18F flortaucipir, followed by a 20 min PET acquisition performed 80 min after injection; 20 min late-uptake PET scans consisted of four 5 min dynamic frames. PET sinograms were reconstructed with OEM into a 256 mm FOV (pixel size 1.0 mm, slice thickness = 3.3 mm). The four individual frames were averaged for analysis. For PiB-PET, subjects were injected with approximately 628 MBq (range, 385–723 MBq) of PiB and after a 40–60 min uptake period a 20 min PiB scan was obtained consisting of four 5 min dynamic frames following a low dose CT transmission scan [59,60]. Median uptake from the GM and WM for specific brain regions was calculated on SPM12 and divided by uptake in the cerebellar crus GM to generate standardized uptake value ratios (SUVRs). All PET images were coregistered to the subject space MPRAGE on DSI Studio using a rigid-body registration type with 200 random search iterations and a mutual iteration cost function. This coregistration established a new set of optimized translation, scaling, shearing, and rotation coordinates to properly adjust the coregistration [52]. We supervised these spatial modifications interactively by visual inspection to avoid misalignment due to extreme neuroanatomical distortions and manually corrected them when appropriate. For all measurements and correlative analysis, right (R) and left (L) ROIs from the Tau-PET and PiB-PET were segmented to specifically capture matching sides of the available neuropathological brain blocks.

### 2.6. Statistical Analysis

Spearman’s rank correlation coefficients (rho) across all BG subregions were performed to assess the relationships between iron staining (PB) and histological tau (AT8) and β-amyloid (6F/3D) in controls, ADNC, and FTLD-tau subjects. Associations between PET SUVRs and PB (%area) were evaluated using Spearman correlation coefficients. GraphPad Prism version 10 (Boston, MA, USA; www.graphpad.com) was used for statistical analysis. A *p*-value (*p* < 0.05) was considered statistically significant. The study cohort consisted of 12 subjects, with each subject contributing up to three anatomically matched basal ganglia measurements from the globus pallidus internus (GPi), globus pallidus externus (GPe), and putamen. Correlation analyses were performed using these regional measurements. Because multiple regional observations were obtained from the same subject, the resulting data are not fully independent. Given the pilot and exploratory nature of this study and the limited number of subjects available with matched imaging and neuropathological assessments, formal modeling of within-subject clustering was not performed. Consequently, all correlation analyses should be interpreted as exploratory and hypothesis-generating. Given the exploratory and hypothesis-generating nature of this pilot study, no formal correction for multiple comparisons was applied. Therefore, all reported *p*-values should be interpreted as uncorrected and considered exploratory. Supplementary descriptive summaries of PB, AT8, and AT8–ferritin colocalization measures stratified by sex were generated. Given the limited sample size and pathological heterogeneity of the cohort, these summaries were presented descriptively without formal statistical comparisons.

## 3. Results

### 3.1. Study Population

The study cohort included 12 neuropathologically characterized cases representing a broad spectrum of tauopathies as well as Alzheimer-related pathology. Of these twelve cases, two did not have any cognitive or motor impairment at the time of death, ages 87 and 100 years, respectively. Both did not have evidence of a pathologically neurodegenerative disease and hence served as controls for this study. Of the other ten cases, two were pathologically diagnosed as having AD neuropathological changes (ADNC) [61]. One case had low-level ADNC (L-AD), and the other had high-level ADNC (H-AD). Eight additional cases had an FTLD-tau diagnosis including four with progressive supranuclear palsy (PSP), one with corticobasal degeneration (CBD), two with globular glial tauopathy (GGT), and one with Pick’s disease (PiD). Specifically, the study included cognitively normal controls (*n* = 2), ADNC (*n* = 2), and FTLD-tau: PSP (*n* = 4), CBD (*n* = 1), GGT (*n* = 2), and PiD (*n* = 1). The sex distribution between the ADNC and FTLD-tau groups were similar, and disease duration showed considerable overlap across diagnostic categories. Participants included both males and females, with ages at death ranging from 61 to 103 years. Among the 10 disease cases, age at symptom onset ranged from 52 to 81 years. PET imaging was performed within a few years of death, enabling close correspondence between in vivo imaging findings and postmortem neuropathological assessments. AD co-pathology varied across the cases, ranging from absent or minimal changes in the controls and several FTLD-tau cases to advanced AD pathology in the high-likelihood ADNC case, with Braak stage VI/VI, Thal phase IV/V, and a maximum ADNC severity score of A3B3C3. Vascular pathology was mild to moderate, although one GGT case demonstrated severe vascular involvement. Overall, the cohort reflects the clinical and pathological heterogeneity characteristic of FTLD tauopathies and Alzheimer’s disease and provides a well-characterized framework for subsequent imaging-pathology analyses. Details of the patient population can be seen in Table 1. Descriptive summaries of PB burden, AT8 pathology, and AT8–ferritin colocalization stratified by sex are provided in Supplementary Table S1. Overall, females demonstrated slightly higher (likely non-significant) mean AT8 burden, whereas PB burden and colocalization measures were comparable between sexes. Because several pathological subgroups were represented by only one or two subjects, individual case-level data are presented whenever possible, and pooled subgroup values should be interpreted only as descriptive summaries.

### 3.2. Neuropathological Findings

Microscopic evaluation of iron aggregates by PB was conducted across all samples (Figure 1a,b). Our quantitative analysis of the percentage of iron per area showed evidence of increased iron burden in both ADNC subjects across all three BG regions. Iron burden on the Pu was greater in the H-AD case than in the L-AD case compared to GPe and GPi regions. Iron burden was greater in all three BG subregions in PSP, PiD, and GGT cases compared to controls (Figure 1c). Tau deposition can be seen in Figure 2a,b. Quantitative analysis demonstrated a larger amount of tau in the H-AD subject in the putamen compared to the other 11 subjects. High levels of AT8 staining (% of the area) were observed across all BG regions in the PSP, CBD, and GGT cases (Figure 2c). PB and AT8 staining were observed to be morphologically similar in the ADNC cases whereby circular-shape iron aggregates were observed (Figure 3a). Isolated neuronal aggregates were observed in controls and the PiD case (Figure 3b,c). Additionally, iron deposits with cellular morphologies resembling glial-like structures were observed together with AT8-positive pathology (insets) in these stainings (Figure 3d–f). Measurements of beta-amyloid staining (Figure 4a,b) showed an increasing staining from the L-AD to the H-AD subject in the putamen, followed by the GPe and GPi. Both PSP subjects had beta-amyloid depositions in the Pu region (Figure 4c).

### 3.3. Fluorescence Immunohistochemical Findings

Double immunofluorescence staining demonstrated variable AT8-positive tau pathology across BG regions and disease groups, with minimal AT8 immunoreactivity in the control cases and progressively greater tau burden in FTLD-tauopathies. Ferritin immunoreactivity was detected in all cases and all regions, although its distribution and extent varied among diseases. Quantitative analysis showed elevated AT8 burden in PSP, CBD, and GGT compared with control tissue, with region-specific differences across the BG. Ferritin-positive area also tended to increase in disease cases, particularly in regions exhibiting greater tau pathology, supporting a relationship between abnormal tau accumulation and altered iron-storage distribution in FTLD-related tauopathies (Figure 5).

Double immunofluorescence staining for 4-repeat tau (RD4) and ferritin demonstrated minimal to absent RD4 immunoreactivity in control and PiD cases, whereas PSP, CBD, and GGT cases showed widespread RD4-positive inclusions throughout the GPi, GPe, and putamen. Ferritin immunoreactivity was detected in all cases and frequently colocalized with RD4-positive structures in PSP, CBD, and GGT, resulting in a distinct yellow signal in merged images. Colocalized RD4/ferritin inclusions were identified in multiple BG regions and confirmed at higher magnification in the inset panels (Figure S1). Among the tauopathy cases, CBD demonstrated the most extensive RD4-positive pathology, with numerous inclusions showing ferritin colocalization. These findings support an association between iron-storage protein accumulation and tau pathology within affected BG regions in primary tauopathies.

AT8–ferritin colocalization, as measured by Pearson’s correlation coefficient (PCC), varied across diagnostic groups and BG regions (Figure 6). Because PCC values were derived from individual subject-level regional measurements, comparisons across pathological subtypes should be interpreted descriptively. The lowest PCC values were observed in the control case (0.01–0.03), indicating minimal spatial overlap between phosphorylated tau and ferritin. Higher colocalization was detected in the H-AD case, with PCC values ranging from 0.12 in the GPi to 0.29 in the putamen. Among the primary tauopathies, PSP cases demonstrated the strongest AT8–ferritin colocalization, particularly in the putamen (PSP-1: PCC = 0.55; PSP-4: PCC = 0.43) and GPe (PSP-1: PCC = 0.43; PSP-4: PCC = 0.29).

Intermediate levels of colocalization were observed in CBD (0.21–0.28) and GGT-1 (0.06–0.32), whereas PiD showed comparatively lower PCC values (0.08–0.22). Across most disease groups, the putamen consistently exhibited the highest degree of AT8–ferritin overlap relative to the GPi and GPe. Overall, these findings indicate greater spatial association between ferritin accumulation and phosphorylated tau pathology in PSP and, to a lesser extent, CBD and GGT, compared with control and PiD cases.

### 3.4. DTI and PET Imaging Findings

DTI data for each subject were quantified as mean diffusivity (MD) within each BG ROI (Figure 7). Compared with controls, FTLD-tau cases demonstrated the greatest MD increases in the globus pallidus externus (GPe) and globus pallidus internus (GPi), with the CBD case exhibiting particularly elevated values in these regions. GGT cases also showed increased MD in the GPi and putamen, whereas the CBD case demonstrated the most prominent MD elevation within the putamen suggesting region-specific microstructural alterations across tauopathy subtypes.

Mean PET SUVR values for the three BG regions are shown in Figure 8a–c. Quantitative analyses revealed consistently higher Tau-PET uptake in both low-AD (L-AD) and high-AD (H-AD) cases across the GPi, GPe, and putamen. Higher flortaucipir uptake was observed in some FTLD-tau cases, including the GGT cases; however, uptake values varied substantially across subjects and overlapped with control values in several non-AD tauopathy cases. However, Tau-PET SUVRs in the CBD, PiD, and GGT cases were comparable to control values and did not demonstrate marked elevations. PiB-PET SUVRs followed a similar overall pattern, with the highest tracer retention observed in the AD cases and a greater increase in the GPe of the PiD case. Overall, PET findings indicate a stronger BG signal in AD compared with the non-AD tauopathies examined in this cohort.

Quantitative DTI- and PET-derived imaging metrics are presented as heat maps in Figure 9a–c.

### 3.5. Histopathological and PET Imaging Correlations

Exploratory analyses identified correlations between iron burden and tau deposition across combined BG regions for the AD group, and to a lesser extent with all FTLD-tau cases combined. These analyses were based on regional measurements obtained from 12 subjects, with each subject contributing up to three anatomically matched basal ganglia observations (GPi, GPe, and putamen). To preserve biological heterogeneity and illustrate the consistency of associations across individual subjects, subject-level regional Spearman correlations are provided in Supplementary Figure S2. Regional PB staining correlated with both AT8 tau pathology and β-amyloid burden across basal ganglia subregions in the AD and FTLD groups (Figure 10a). There was a modest correlation between PB and Tau-PET and PiB-PET uptake, which was stronger in the ADNC group than in the FTLD-tau group. Within this small cohort, regional iron burden in the ADNC cases was positively correlated with Tau-PET uptake (ρ = 0.38, *p* = 0.011) (Figure 10b). Similarly, in the FTLD group, iron burden correlated with Tau-PET uptake (ρ = 0.58, *p* < 0.0001) and PiB-PET uptake (ρ = 0.53, *p* < 0.0001).

## 4. Discussion

Our study combined quantitative neuropathology, fluorescence immunohistochemistry, diffusion MRI, and molecular PET imaging to investigate the relationship between iron accumulation and protein aggregation within BG regions affected by ADNC and FTLD-tau. Across multiple complementary modalities, exploratory analyses revealed consistent patterns of association between iron burden and pathological protein deposition.

Specifically, regional iron accumulation demonstrated significant associations with both tau and β-amyloid pathology, while ferritin frequently colocalized with pathological tau aggregates in disease-affected regions. These findings are consistent with a possible association between altered iron homeostasis and neurodegenerative proteinopathies, although the present study was not designed to establish biological mechanisms or causal relationships.

Iron accumulation within BG structures has been extensively reported in AD by both neuropathological and neuroimaging studies [63,64]. However, less is known regarding the regional distribution of iron in FTLD-tauopathies. In the present study, iron deposition was heterogeneous across both diseases and BG subregions.

The Pu regions consistently demonstrated the highest iron burden, particularly in the high-likelihood AD case, whereas PSP cases exhibited more widespread iron accumulation involving the Pu, GPi, and GPe subregions.

These findings suggest that iron deposition is neither uniform across diseases nor across anatomical regions but instead follows disease-specific patterns that may reflect differing pathogenic mechanisms. Although the biological significance of these regional differences remains uncertain, the substantial iron burden observed in PSP supports previous reports that disturbances in iron regulation may be a prominent feature of selected FTLD-tau syndromes.

Experimental and neuropathological studies have implicated iron in multiple pathways that may promote tau pathology, including oxidative stress, kinase activation, impaired phosphatase activity, and direct facilitation of tau aggregation [65,66,67]. Conversely, tau pathology itself may further disrupt iron homeostasis, suggesting a potentially bidirectional relationship between iron accumulation and protein aggregation [23,29,68]. Although the present study was not designed to establish causality, the observed associations between iron deposition, tau pathology, and PET biomarkers are consistent with these proposed mechanisms and are consistent with the hypothesis that altered iron metabolism may be associated with neurodegenerative processes in both ADNC and FTLD-tau.

One of the principal strengths of this study is the availability of matched antemortem imaging and postmortem neuropathological assessments. Few investigations have directly compared histopathological measures of iron accumulation with corresponding Tau-PET and PiB-PET biomarkers in the same subjects. We observed significant correlations between regional iron burden and both Tau-PET and PiB-PET uptake in ADNC and FTLD-tau groups, providing preliminary evidence that in vivo molecular imaging may capture aspects of the pathological processes occurring within iron-rich BG structures. While these associations were stronger in the AD group, significant relationships were also identified in FTLD-tau subjects, supporting the relevance of iron accumulation beyond Alzheimer-related pathology. Given the substantial differences in spatial resolution between microscopic pathology and macroscopic PET measures, the observed correlations should be interpreted as evidence of biological association rather than direct pathogenic interactions. An additional consideration is that partial-volume correction was not performed. Several disorders included in this cohort, particularly PSP, CBD, and GGT, are associated with regional neurodegeneration and tissue loss that may influence PET signal measurements within relatively small basal ganglia structures. Consequently, regional SUVR values may be affected by partial-volume effects and signal spill-over from adjacent tissues, potentially contributing to underestimation or overestimation of tracer uptake. Therefore, small regional differences in PET measurements should be interpreted cautiously and should not be overinterpreted as precise quantitative differences in underlying pathology. Nevertheless, the consistency of these findings across pathology and imaging modalities is consistent with a relationship between iron dysregulation and protein aggregation. However, the present data cannot determine whether these processes are causally related. The interpretation of these Tau-PET findings warrants caution, particularly in FTLD-tau. Flortaucipir was originally developed to detect Alzheimer-type paired helical filament tau and may exhibit variable sensitivity across non-AD tauopathies. As a result, tracer uptake in FTLD-tau does not necessarily provide a direct quantitative measure of regional tau burden and may underestimate pathology in specific tauopathy subtypes. Therefore, the correlations observed between iron burden and Tau-PET uptake should be interpreted as exploratory associations between neuropathological and imaging measures rather than evidence that PET signal directly reflects the magnitude of underlying tau pathology. The interpretation of the PiB-PET findings also warrants caution. The FTLD-tau cohort exhibited substantial variability in Alzheimer-related co-pathology, including differences in Braak stage, Thal phase, and ADNC classification. Consequently, the observed association between iron burden and PiB-PET uptake within FTLD-tau cases may partly reflect concomitant Alzheimer-type pathology rather than a biological feature intrinsic to FTLD-tau itself. Given the limited sample size and inability to adjust for these factors, the PiB-PET correlations should be interpreted as exploratory associations within a heterogeneous pathological cohort rather than evidence that amyloid accumulation is a characteristic feature of FTLD-tau.

A novel aspect of this study was the demonstration of tau-ferritin colocalization using fluorescence immunohistochemistry. Ferritin immunoreactivity was present across all cases and BG regions, consistent with its physiological role as the primary intracellular iron-storage protein. Disease cases, however, exhibited greater overlap between ferritin-positive structures and pathological tau aggregates than controls. Frequent AT8–ferritin colocalization was observed in PSP, CBD, GGT, and H-AD cases, indicating that abnormal iron storage often occurs within the same cellular environments that harbor pathological tau. The RD4 immunofluorescence findings further extend this observation. Ferritin frequently colocalized with RD4-positive inclusions in PSP, CBD, and GGT cases, suggesting that altered iron handling may be associated with 4-repeat tau pathology in these individual cases rather than simply representing a nonspecific consequence of neurodegeneration. Together, these findings are consistent with associations among histological markers of iron biology, pathological protein deposition, and neurodegenerative disease.

It is important to emphasize that the iron-related measures evaluated in this study reflect distinct aspects of iron biology and should not be considered interchangeable. PB staining identifies ferric iron present within certain iron-containing deposits but does not capture all biologically relevant forms of tissue iron. In contrast, ferritin immunoreactivity reflects the distribution of an iron-storage protein and does not directly quantify iron concentration. Therefore, neither PB staining nor ferritin immunostaining alone provides a comprehensive assessment of cellular iron homeostasis. Rather, the observed associations among PB staining, ferritin immunoreactivity, tau pathology, and imaging biomarkers should be interpreted as reflecting relationships among complementary markers of iron biology rather than direct measures of global iron dysregulation.

Interestingly, the relationship between iron and tau pathology differed across FTLD-tau subtypes. Among the cases included in this cohort, variability was observed in the relationship between iron-related measures and tau pathology across FTLD-tau syndromes. However, because several pathological subgroups were represented by only one or two subjects, these observations should be interpreted cautiously and not as evidence of disease-specific effects. Overall, the findings highlight the biological heterogeneity of FTLD-tau and support the need for larger pathology-specific studies to further investigate the relationship between iron-related markers and tau pathology. Importantly, considerable variability was also observed among subjects within the same pathological subgroup, particularly among PSP and GGT cases. This heterogeneity is further illustrated by the subject-level regional correlation analyses presented in Supplementary Figure S2, which show the direction and magnitude of PB-pathology/PET associations for individual cases. For this reason, individual case-level findings were retained and presented throughout the manuscript whenever feasible, including the neuropathological and colocalization analyses. Although pooled PSP and GGT values were provided in selected summary tables and visualizations to facilitate descriptive interpretation, these averages should not be viewed as evidence of homogeneous disease-specific effects. Rather, they are intended to complement the individual subject data and highlight broader trends while preserving recognition of the substantial biological heterogeneity present within FTLD-tau syndromes.

Our neuropathological observations were further supported by the morphological characteristics of iron-positive inclusions. Iron accumulation frequently appeared within glial-like cellular structures and often resembled tau-positive lesions previously described in PSP and related tauopathies [39]. Prior studies have suggested that glial cells may play a significant role in regulating iron homeostasis by increasing iron sequestration during pathological conditions [69,70]. The frequent colocalization of ferritin with both AT8-positive and RD4-positive tau pathology observed in the present study raises the possibility that alterations in iron storage occur within the same cellular environments in which pathological tau inclusions are observed. However, the specific cellular identity of these structures cannot be determined from morphology alone and was not confirmed using cell-type-specific markers. Although mechanistic conclusions cannot be drawn from the current data, these observations are consistent with an association between iron dysregulation and cellular alterations observed in FTLD-tau.

In addition to the molecular imaging findings, diffusion MRI revealed increased mean diffusivity within BG regions of several FTLD-tau cases, particularly within iron-rich structures such as the putamen and globus pallidus. Similar observations have been reported previously in diffusion MRI studies of FTLD-related disorders [71,72]. Increased diffusivity may reflect disruption of tissue organization resulting from neuronal loss, gliosis, or degeneration of local cellular networks. Although DTI does not directly measure iron accumulation, the regional correspondence between elevated diffusivity, iron deposition, and tau pathology suggests that elevated diffusivity, iron deposition, and tau pathology may occur together within affected BG circuits. These findings therefore provide complementary evidence that iron-rich regions are also characterized by measurable alterations in tissue integrity in vivo.

Several limitations should be considered when interpreting these findings. First, the sample size was small given that all subjects were required to have matched antemortem DTI MRI, Tau-PET, PiB-PET, and postmortem neuropathological evaluation. Consequently, statistical power was limited, especially when we separated the analyses by group. Therefore, the findings should be regarded as exploratory and hypothesis-generating rather than hypothesis testing. Because all analyses were observational, cross-sectional, and correlation-based, the present study cannot establish whether iron accumulation precedes, follows, or occurs independently of tau pathology, nor can it determine causal relationships between these processes. Additionally, multiple correlation analyses were performed without formal correction for multiple comparisons. Instead, correlation analyses were performed using regional measurements from multiple basal ganglia structures within the same subjects. Because these observations are not fully independent, the effective sample size may be smaller than the number of regional measurements included in the analyses. Specifically, each participant contributed measurements from up to three basal ganglia subregions (GPi, GPe, and putamen), resulting in within-subject clustering that was not formally modeled. As a result, the reported *p*-values should be interpreted cautiously and primarily as exploratory indicators of association rather than confirmatory statistical evidence. Furthermore, because multiple correlation analyses were performed without formal correction for multiple comparisons, the possibility of type I error should be considered when interpreting statistically significant findings.

Given the pilot nature of the study and limited sample size, the analyses were intended to identify potential associations rather than provide confirmatory statistical evidence. Consequently, the findings should be interpreted cautiously and require validation in larger independent cohorts. In addition to its small size, the cohort was derived from a single tertiary academic center and consisted exclusively of research-enrolled individuals who underwent autopsy and had matched antemortem imaging and postmortem neuropathological assessments. As a result, the study population represents a highly selected sampling frame that may not fully reflect the broader clinical and demographic spectrum of patients with FTLD-tau and related disorders encountered in the general population. This limitation is distinct from statistical underpowering. Specifically, restricted cohort representativeness and the inherent biological heterogeneity of neurodegenerative diseases constitute separate and potentially compounding threats to generalizability.

While the present design enabled detailed multimodal imaging-pathology correlations, future multi-center studies with more diverse populations will be necessary to determine the extent to which these findings generalize across different demographic, clinical, and pathological settings. The small sample size precluded adjustment for potentially important confounders such as age, disease duration, sex, co-pathology burden, and medication exposure. In particular, variability in Alzheimer-type co-pathology, including differences in Braak stage, Thal phase, and ADNC burden, may have influenced both neuropathological and PET imaging measurements. Because adjustment for these factors was not feasible, the observed associations involving PiB-PET uptake, especially within the FTLD-tau cohort, should not be interpreted as evidence that amyloid accumulation is an intrinsic characteristic of FTLD-tau. In particular, biological sex may contribute to variability in iron deposition, tau pathology, and imaging biomarkers independent of disease category. Although the present cohort was not sufficiently powered to support meaningful sex-stratified analyses, sex-related effects should be considered a potential unexamined source of variance and warrant investigation in larger cohorts. Furthermore, substantial variability was observed among individuals within the same pathological subgroup, supporting the importance of preserving case-level observations in studies of heterogeneous neurodegenerative syndromes [73].

Second, the cohort included multiple pathological diagnoses, limiting disease-specific conclusions and contributing to overall pathological heterogeneity. Furthermore, pooled PSP and GGT summaries were included solely for descriptive visualization purposes and should not be interpreted as evidence of disease-level effects, particularly given the substantial interindividual variability observed within these groups. Several pathological subgroups were represented by only one or two subjects, including CBD, PiD, and ADNC cases. Consequently, observations within these subgroups should be interpreted as individual case-level findings rather than evidence of disease-specific patterns. Third, quantitative comparisons between histopathological measurements and PET SUVRs are inherently constrained by substantial differences in spatial resolution between microscopic tissue analyses and macroscopic neuroimaging techniques. Furthermore, flortaucipir PET has recognized limitations in non-Alzheimer tauopathies. Because tracer binding characteristics differ across tau conformations and isoforms, regional flortaucipir uptake may not accurately reflect the absolute burden of tau pathology in FTLD-tau syndromes. Consequently, associations between Tau-PET measurements and histopathological findings should be interpreted cautiously, particularly when comparing different pathological subtypes.

An additional limitation relates to the PET analyses. Partial-volume correction was not performed, and therefore PET signal measurements within small basal ganglia structures may be substantially influenced by partial-volume effects and spill-over from adjacent tissues. This issue is particularly important in the present cohort because several disorders examined, including PSP, CBD, and GGT, are characterized by regional neurodegeneration and tissue loss that may alter the accuracy of PET signal quantification. As a result, regional tracer uptake values may not precisely reflect the underlying pathological burden within individual basal ganglia subregions. These effects may have contributed to both underestimation and overestimation of regional SUVR measurements and may also have influenced the magnitude of the observed PET-pathology correlations. Therefore, small regional differences in PET uptake should be interpreted cautiously and should not be overinterpreted as precise measures of underlying neuropathology. Consequently, the observed associations between PET biomarkers and histopathological measures should be interpreted with appropriate caution. Also, ABC scores provide a global summary of disease severity and do not necessarily reflect the precise burden of tau or β-amyloid pathology within the BG. Another limitation is that the study evaluated selected histological markers related to iron biology rather than providing a comprehensive assessment of iron homeostasis. PB staining primarily detects a subset of ferric iron deposits, whereas ferritin immunoreactivity reflects expression of an iron-storage protein. Consequently, these measures provide complementary but incomplete information regarding iron metabolism, and the present findings should not be interpreted as a comprehensive characterization of cellular iron regulation or dysregulation.

An additional limitation relates to the interpretation of cellular morphology. Although iron-positive structures frequently exhibited glial-like or neuronal-like morphological features, cell-type-specific immunohistochemical markers were not employed. Therefore, the cellular identity of these structures could not be definitively established, and references to glial-like or neuronal-like morphology should be interpreted descriptively rather than as definitive cellular classifications. Fourth, the study focused exclusively on BG regions because of their recognized susceptibility to iron accumulation and the availability of corresponding neuropathological material; therefore, it remains unknown whether similar relationships exist throughout cortical and other subcortical regions. Furthermore, the cross-sectional nature of the study prevents determination of whether iron accumulation precedes, follows, or occurs concurrently with tau and β-amyloid deposition. Future studies incorporating larger pathology-specific cohorts, additional brain regions, complementary histological and biochemical measures of iron homeostasis, and longitudinal multimodal imaging-pathology approaches will be necessary to clarify the temporal and mechanistic relationships between iron accumulation, tau pathology, and β-amyloid deposition.

Taken together, these findings support a consistent association between iron accumulation and pathological protein deposition across multiple complementary modalities. Quantitative histopathology, tau–ferritin colocalization analyses, diffusion MRI, and molecular PET imaging each pointed toward a relationship between altered iron homeostasis and tau pathology within BG regions affected by ADNC and FTLD-tau. Notably, PSP and other 4-repeat tauopathy cases in this cohort demonstrated higher levels of iron burden, tau pathology, and ferritin colocalization, suggesting differing patterns of association across tauopathy subtypes. However, these observations are exploratory and should not be interpreted as evidence of mechanistic differences among diseases. While these observations remain exploratory, they provide a biologically plausible framework linking iron dysregulation to protein aggregation and support the integration of neuropathological and in vivo imaging approaches for studying neurodegenerative disease.

## 5. Conclusions

This pilot study provides preliminary evidence of associations between iron burden and tau-related pathology in FTLD-tau and ADNC. The observation of iron accumulation within glial-like structures and its spatial relationship with tau pathology is consistent with a potential association between altered iron homeostasis and neurodegenerative pathology; however, these findings remain exploratory and hypothesis-generating. Moreover, the interpretation of Tau-PET findings should consider the known limitations of flortaucipir in non-AD tauopathies. Future studies using larger pathology-specific cohorts and longitudinal multimodal imaging-pathology approaches are needed to validate these preliminary findings and help clarify the temporal relationship between iron accumulation, tau pathology, and β-amyloid deposition.

## Figures and Tables

**Figure 1:**
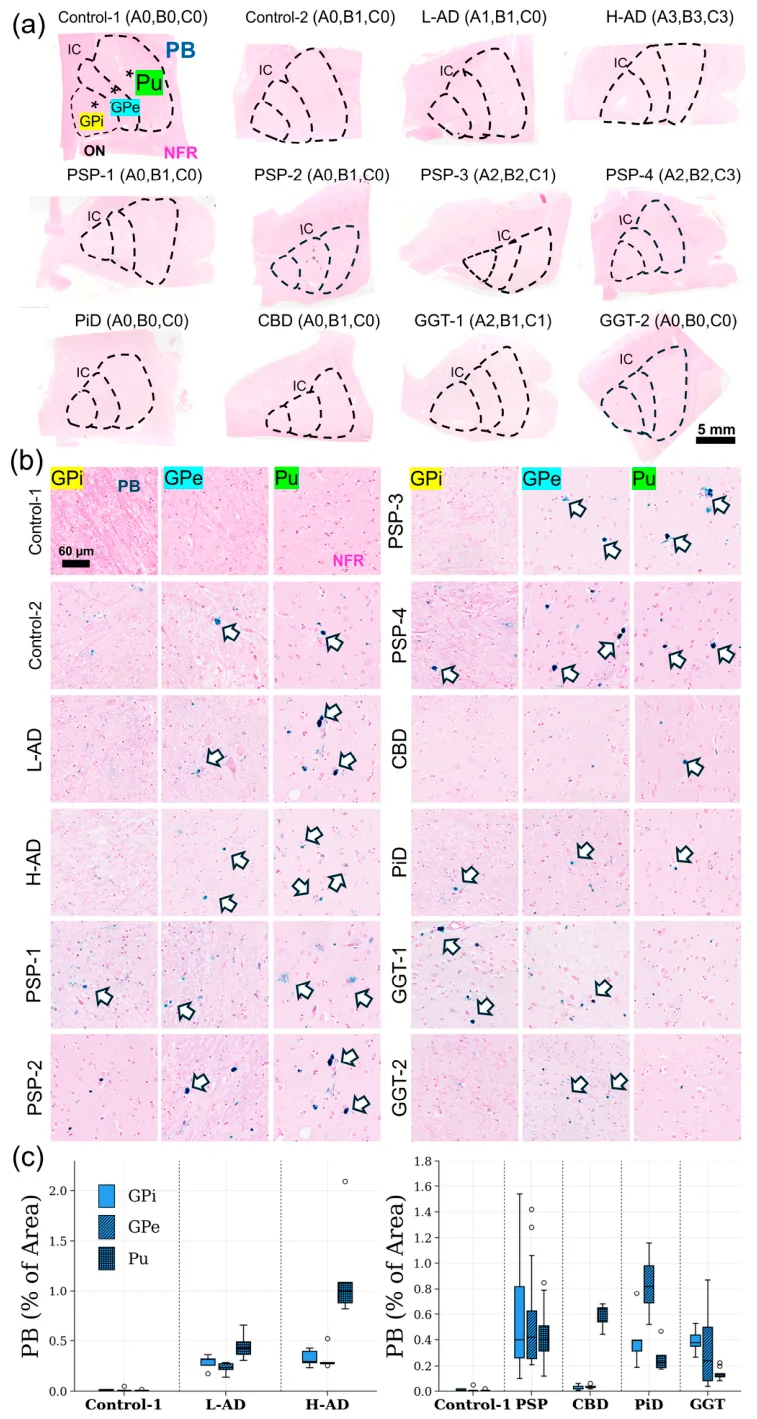
Iron staining across basal ganglia substructures. (**a**) Histopathological segmentation of basal ganglia (BG) subregions from AD and FTLD subjects from paraffin—paraformaldehyde-fixed brain block specimens obtained with Prussian Blue (PB) and counterstained with Fast Nuclear Red (FNR). (**b**) Augmented view from Figure 3 of histopathological images of three different BG subregions. (**c**) Quantitative calculations of PB across each subject and sub region. Abbreviations: GPi, globus pallidum internus; GPe, globus pallidum externus; Pu, putamen; ON, optical nerve; IC, internal capsule; H-AD, high-likelihood Alzheimer’s disease; PSP-1, progressive supranuclear palsy Richardson syndrome (PSP-RS); PSP-2, PSP-3, and PSP-4, progressive supranuclear palsy with speech/language variant (PSP-SL); CBD, corticobasal degeneration; PiD, Pick’s disease; GGT, globular glial tauopathy; PB, Prussian blue; NFR, nuclear fast red. Asterisks (*) identify the visually defined GPi, GPe, and Pu subregions, whereas arrows indicate representative Prussian blue-positive iron deposits/inclusions.

**Figure 2:**
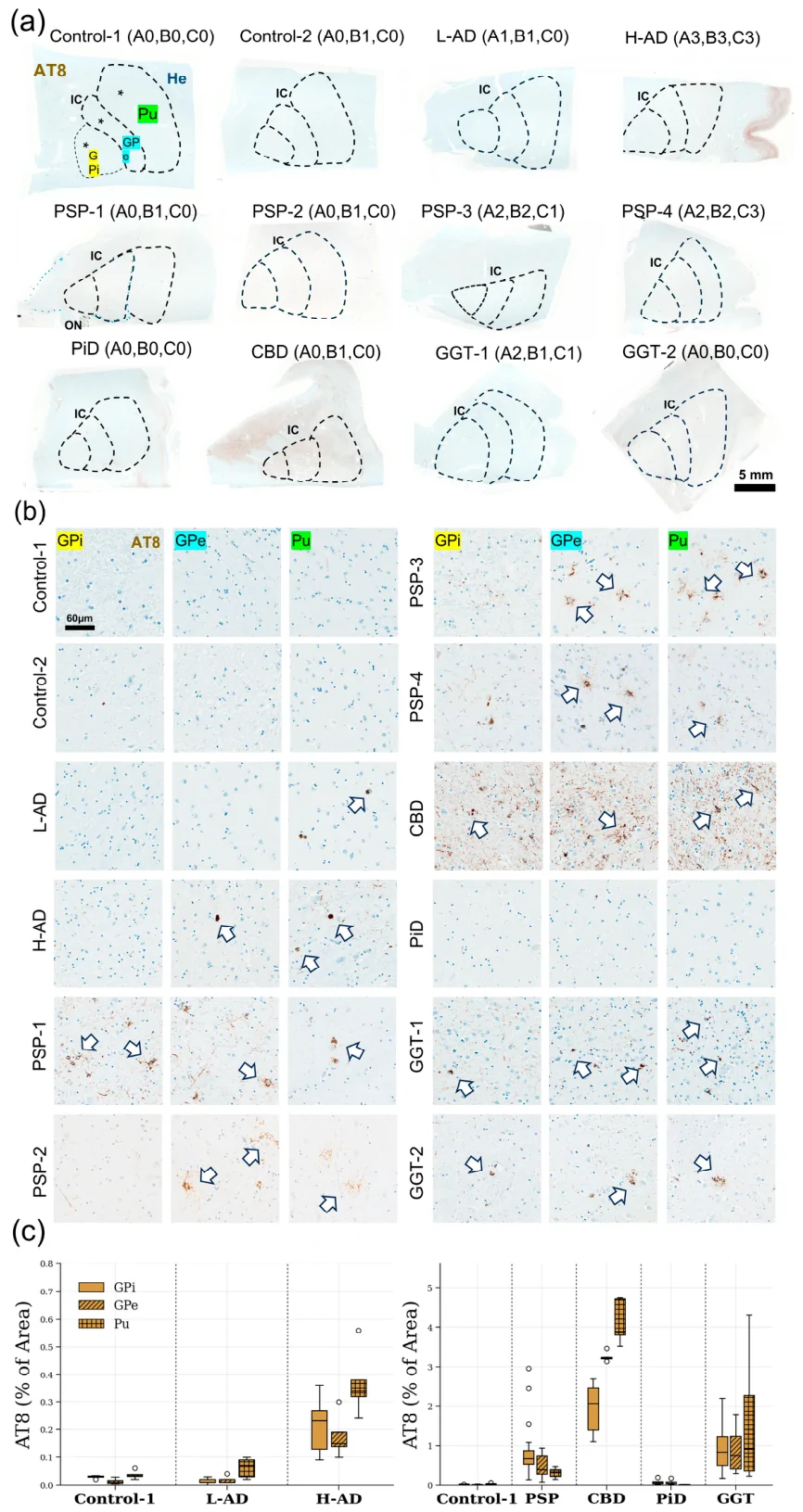
Histological samples from BG stained with AT8. (**a**) Histological preparations are presented, and subregions of interest are displayed for comparison. (**b**) Increasing AT8 immunoreactivity was observed in PSP-1 (PSP-RS), PSP-2 to PSP-4 (PSP-SL), and the CBD case, with lower levels in the GGT case (white arrows). (**c**) Quantitative assessment of phosphorylated AT8 tau across each subject GPi, GPe, and Pu in the AD subgroups (**left**) and FTLD subgroups (**right**). Abbreviations: GPi, globus pallidum internus; GPe, globus pallidum externus; Pu, putamen; ON, optical nerve; IC, internal capsule; H-AD, high-likelihood Alzheimer’s disease; PSP-RS, progressive supranuclear palsy Richardson syndrome; PSP-SL, progressive supranuclear palsy with speech/language variant; CBD, corticobasal degeneration; PiD, Pick’s disease; GGT, globular glial tauopathy; Asterisks identify the visually defined GPi, GPe, and Pu subregions.

**Figure 3:**
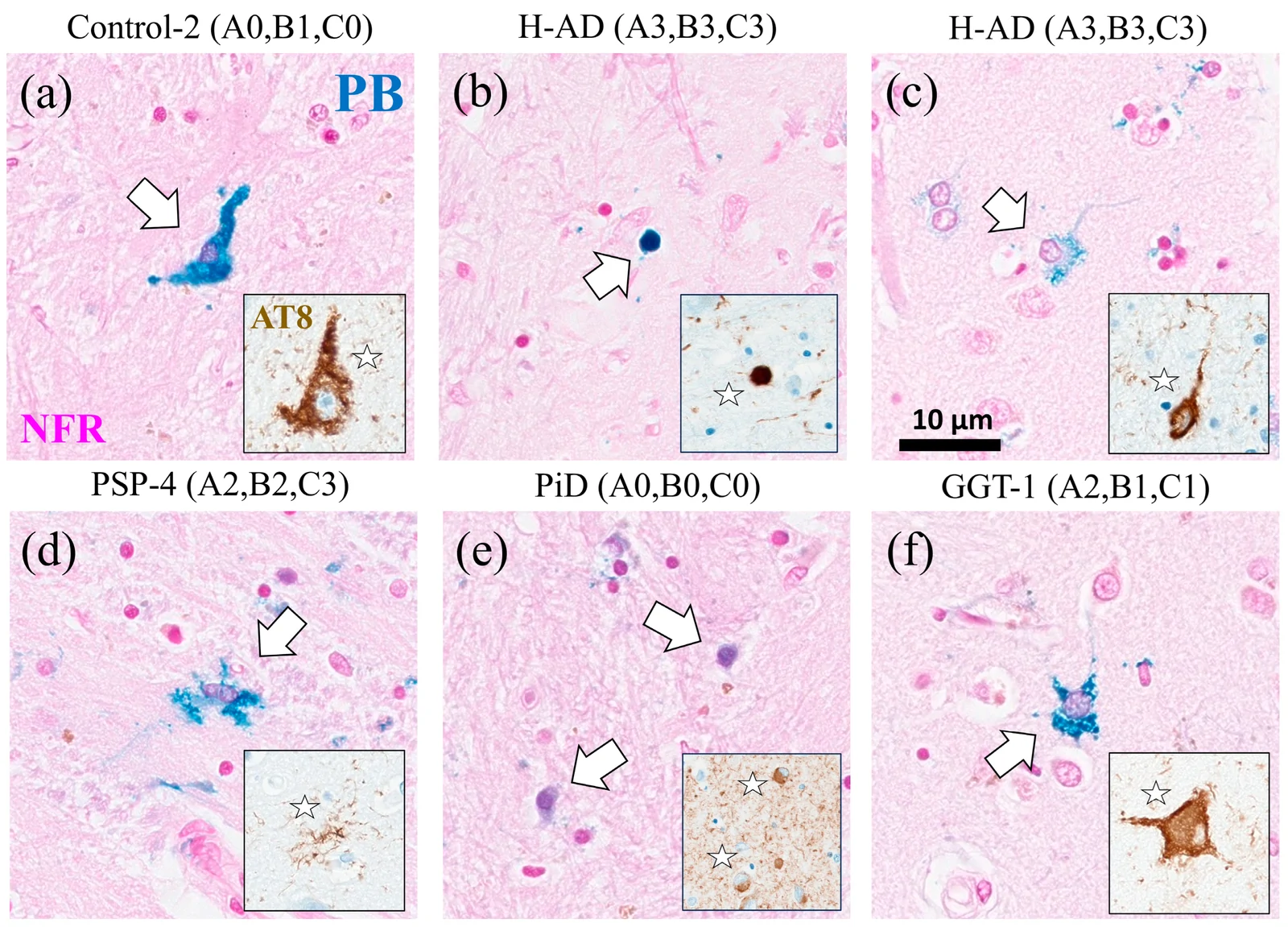
Iron-positive inclusions exhibit morphological features resembling tau pathology. (**a**) A cellular Prussian blue (PB)-positive iron inclusion (white arrow) identified in the putamen (Pu) of an extremely elderly control subject. (**b**) Spherical iron-positive inclusions (white arrow) were observed in both Alzheimer’s disease (AD) and frontotemporal lobar degeneration (FTLD) cases, frequently appearing within the extracellular compartment. (**c**) Iron accumulation detected within a cellular structure in a high-likelihood AD (H-AD) case (white arrow). (**d**–**f**) Representative examples of iron aggregation in distinct cellular morphologies from PSP, PiD, and GGT cases (white arrows). Notably, several iron-positive inclusions displayed morphological characteristics similar to tau-positive lesions observed in corresponding cell types (white stars), as illustrated in the lower-right inset panels. These findings demonstrate spatial co-occurrence between iron-positive structures and tau pathology across diverse neurodegenerative tauopathies. Acronyms: PB, Prussian blue; NFR, Nuclear Fast Red; Pu, putamen; AD, Alzheimer’s disease; FTLD, frontotemporal lobar degeneration; PSP, progressive supranuclear palsy; PiD, Pick’s disease; GGT, globular glial tauopathy.

**Figure 4:**
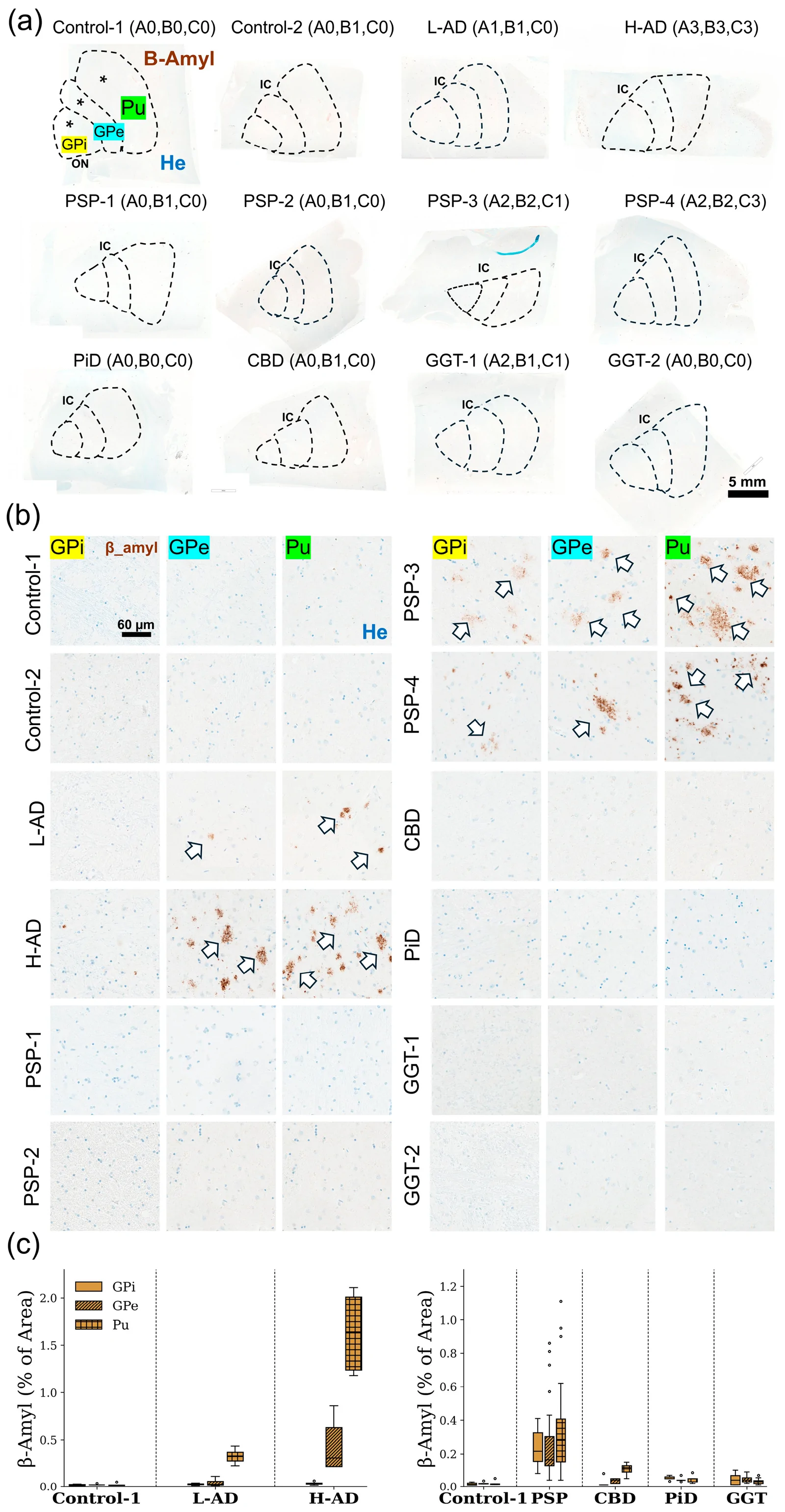
β-Amyloid Pathology in BG Regions Across Alzheimer’s Disease and FTLD Cases. (**a**) Representative BG sections immunostained for β-amyloid, illustrating the regional distribution of amyloid pathology across diagnostic groups (white arrows). (**b**) Higher-magnification views of the globus pallidus internus (GPi), globus pallidus externus (GPe), putamen (Pu), and caudate nucleus demonstrate increased β-amyloid burden in intermediate- and high-likelihood Alzheimer’s disease (L-AD and H-AD) cases. Focal β-amyloid deposition was also observed in the GPi, GPe, and Pu of PSP-3 and PSP-4. (**c**) Quantitative assessment of β-amyloid burden within regions of interest (ROIs) confirmed greater amyloid accumulation in AD compared with FTLD cases (white arrows), with only limited deposition detected in selected FTLD subjects. These findings suggest that β-amyloid pathology is associated with AD but may occur at lower levels in specific FTLD subtypes. Abbreviations: GPi, globus pallidus internus; GPe, globus pallidus externus; Pu, putamen; ROI, region of interest; AD, Alzheimer’s disease; L-AD, low-likelihood Alzheimer’s disease; H-AD, high-likelihood Alzheimer’s disease; FTLD, frontotemporal lobar degeneration; PSP-SL, progressive supranuclear palsy–speech/language variant. Scale bars as indicated; Asterisks identify the visually defined GPi, GPe, and Pu subregions.

**Figure 5:**
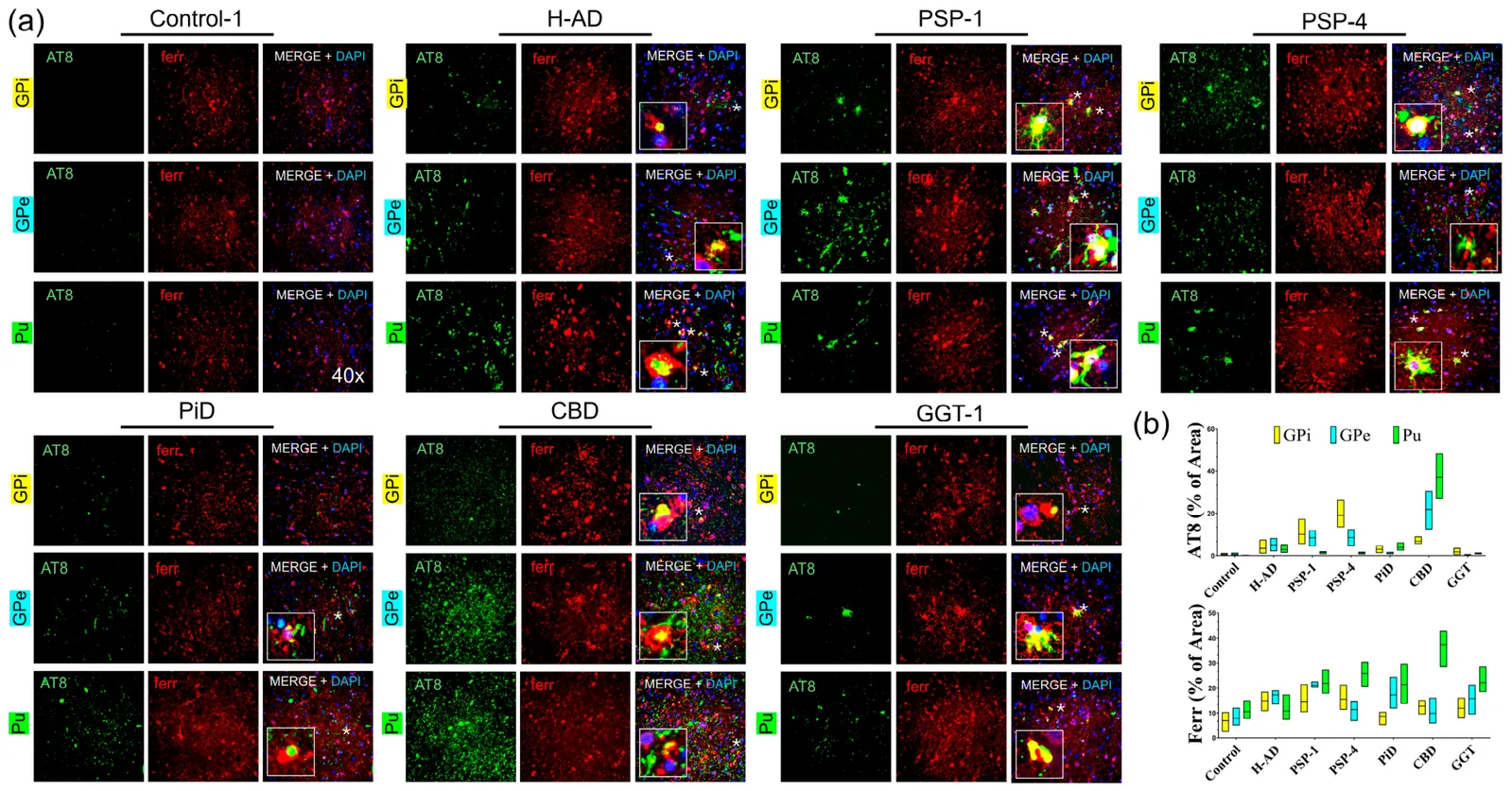
Double immunofluorescence staining for phosphorylated tau (AT8, green) and ferritin (red) in BG regions from control and neurodegenerative disease cases, including high-likelihood Alzheimer’s disease (H-AD), progressive supranuclear palsy (PSP), Pick’s disease (PiD), corticobasal degeneration (CBD), and globular glial tauopathy (GGT). (**a**) Representative images from the globus pallidus internus (GPi), globus pallidus externus (GPe), and putamen (Pu). Merged images include DAPI nuclear counterstaining (blue). Areas showing colocalization of AT8 and ferritin are visualized as yellow/orange signal and are highlighted by white asterisks; corresponding higher-magnification views are shown in the inset panels. Pronounced AT8 pathology and tau–ferritin colocalization were observed in tauopathy cases, whereas control tissue exhibited minimal AT8 immunoreactivity. (**b**) Quantification of AT8-positive area (%) (**upper** panel) and ferritin-positive area (%) (**lower** panel) across the three BG regions. Images were acquired at 40× magnification. Abbreviations: GPi, globus pallidus internus; GPe, globus pallidus externus; Pu, putamen; AT8, phosphorylated tau.

**Figure 6:**
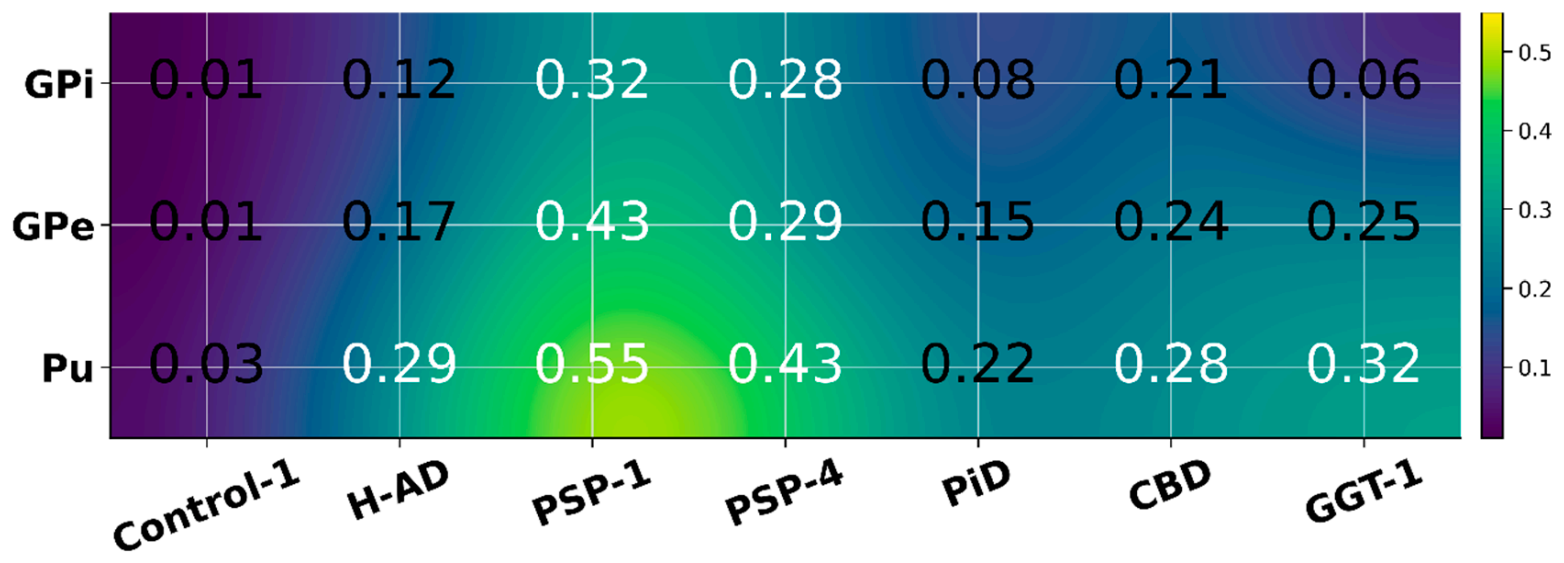
Regional Tau–Ferritin Colocalization in BG Tauopathies. Footnote: Representative mean Pearson’s colocalization coefficients (PCC) between AT8-immunoreactive tau pathology and ferritin immunostaining measured by confocal microscopy across selected basal ganglia (BG) regions of interest (ROIs), including the globus pallidus internus (GPi), globus pallidus externus (GPe), and putamen (Pu). Warmer colors indicate higher PCC values and cooler colors indicate lower PCC values. For each subject and BG subregion, PCC values represent the average of four independently analyzed microscopic fields, acquired using identical imaging parameters and preprocessing procedures, as described in Section 2.3. Higher PCC values indicate greater spatial overlap between tau aggregates and ferritin-positive structures. Across all regions, the strongest colocalization was observed in PSP cases, particularly within the putamen, whereas lower colocalization values were observed in control and AD cases. Values are presented as representative regional averages. Because several pathological categories were represented by a single subject, PCC values should be interpreted as individual case-level measurements rather than disease-specific group estimates. Abbreviations: PCC, Pearson’s colocalization coefficient; ROI, region of interest; BG, basal ganglia; GPi, globus pallidus internus; GPe, globus pallidus externus; Pu, putamen; AD, Alzheimer’s disease; PSP, progressive supranuclear palsy; CBD, corticobasal degeneration; PiD, Pick’s disease; GGT, globular glial tauopathy.

**Figure 7:**
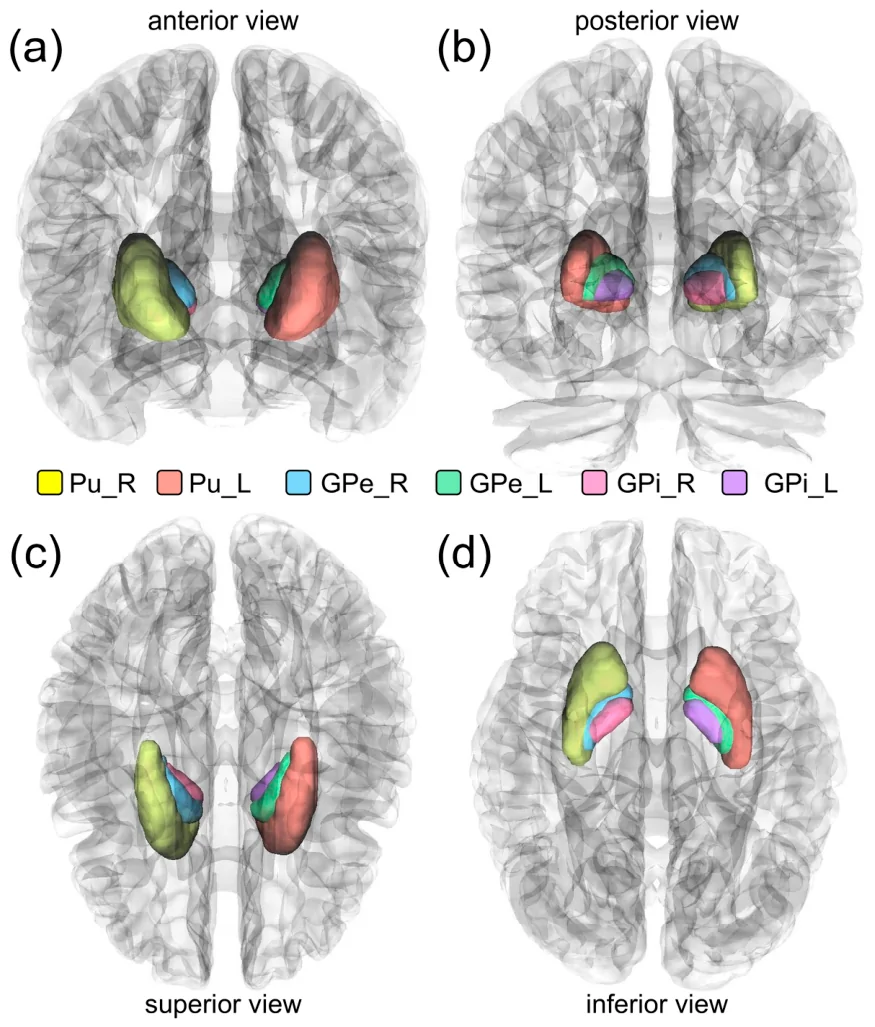
Premortem diffusion tensor imaging (DTI) and automated segmentation of subcortical regions of interest. Three-dimensional renderings of the segmented structures are displayed in anterior (**a**), posterior (**b**)**,** superior (**c**), and inferior (**d**) orientations to illustrate their spatial distribution. Mean diffusivity (MD) values were obtained in common space from the hemisphere corresponding to the neuropathologically evaluated side. Abbreviations: R, right; L, left; GPi, globus pallidus internus; GPe, globus pallidus externus; Pu, putamen.

**Figure 8:**
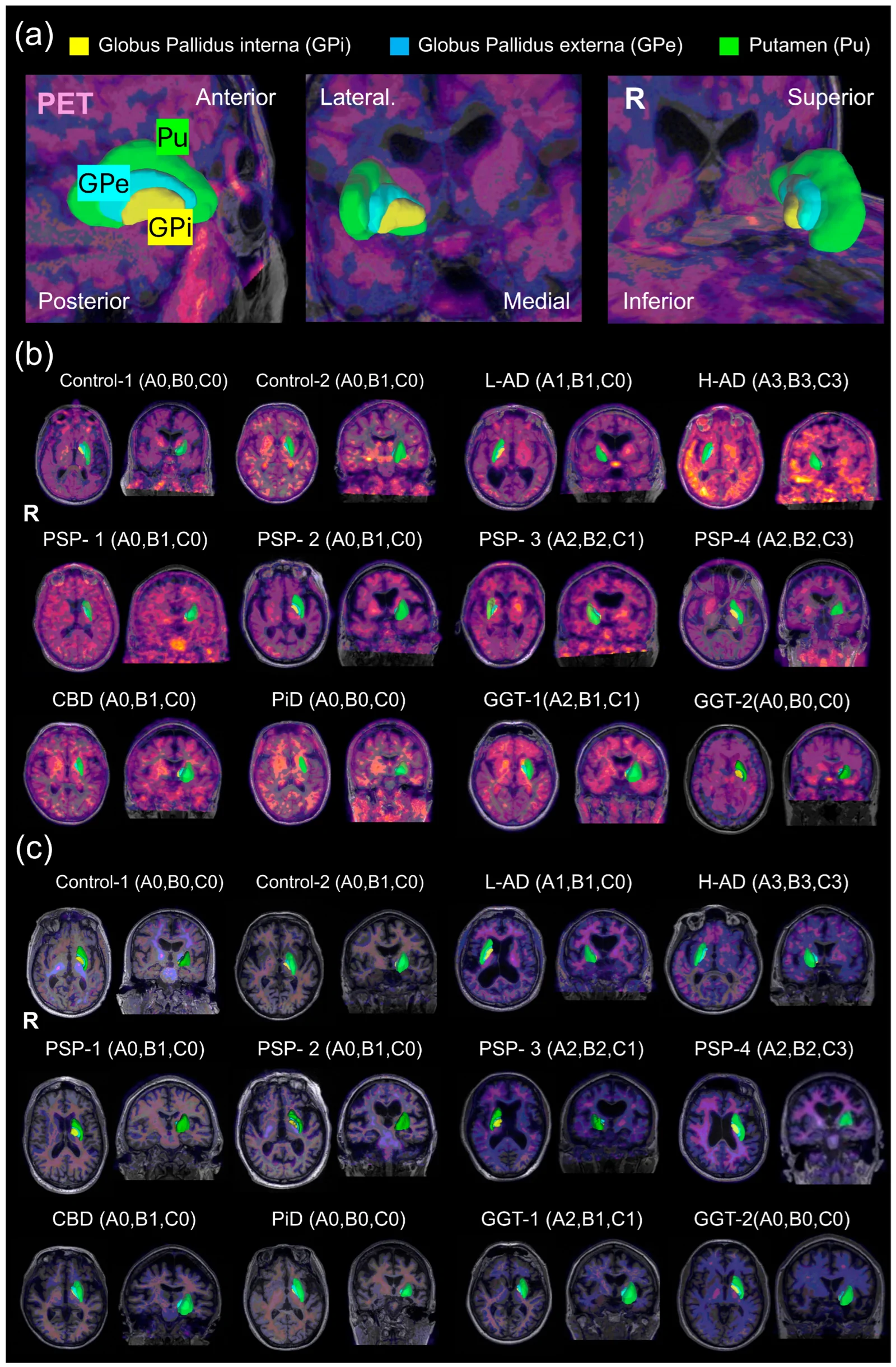
BG segmentation and PET imaging coregistration. (**a**) Structural MRI (MPRAGE) coregistered with PET imaging showing automated segmentation of BG subregions, including the globus pallidus internus (GPi), globus pallidus externus (GPe), and putamen (Pu). (**b**) MRI and tau PET (^18^F-flortaucipir) coregistration, with hemispheric regions selected to match the side available for neuropathologic evaluation. (**c**) MRI and PiB-PET coregistration depicting β-amyloid burden in representative AD and FTLD cases, together with their corresponding ADNC scores. Abbreviations: R, right; L, left; BG, basal ganglia; GPi, globus pallidus internus; GPe, globus pallidus externus; Pu, putamen.

**Figure 9:**
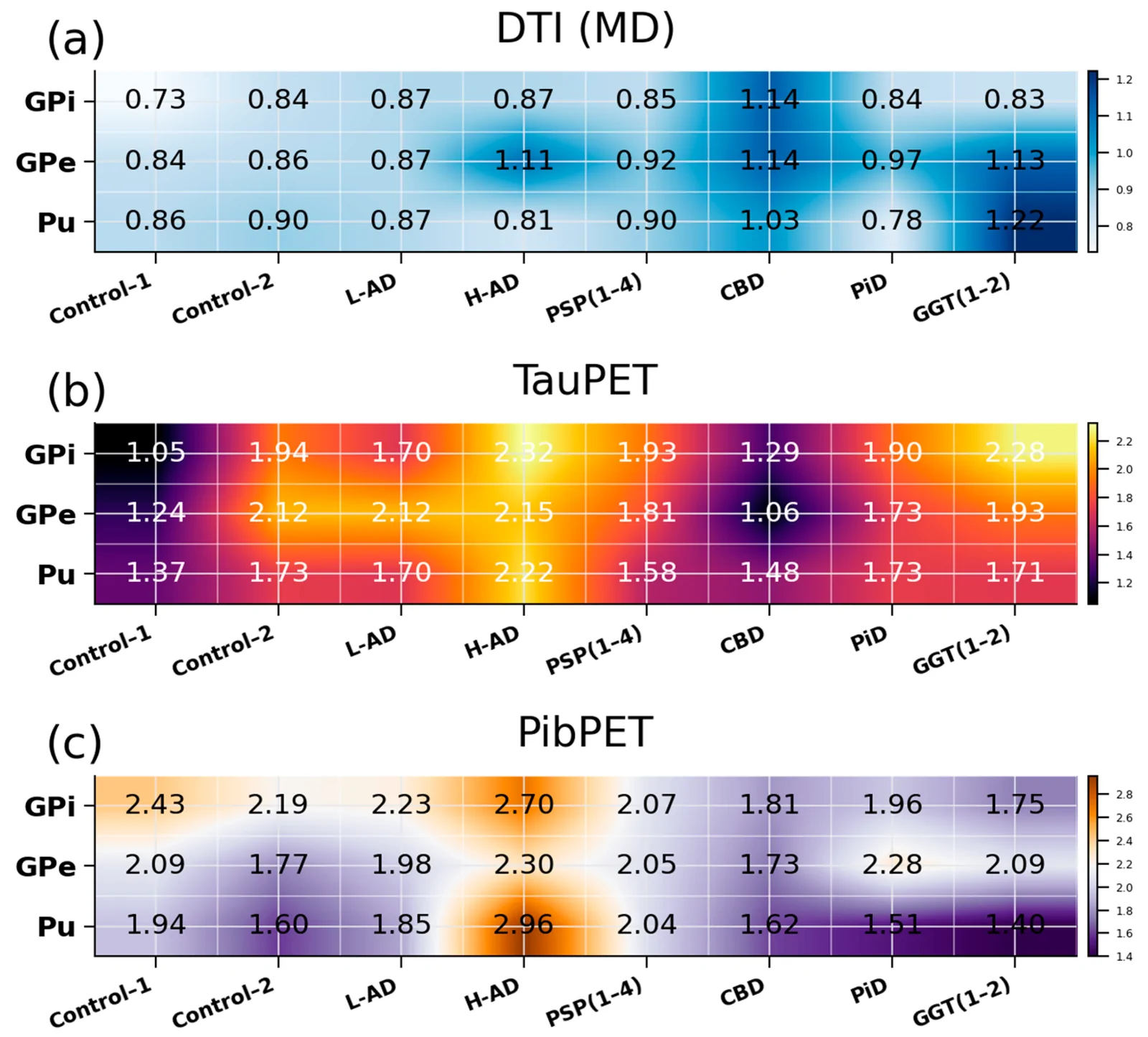
Multimodal Imaging Metrics in BG Regions. Heat map color intensity reflects the relative magnitude of the measured values. Diffusion tensor imaging (DTI)-derived mean diffusivity (MD) values are reported in units of ×10^−4^ mm^2^/s (**a**). TauPET (**b**) and PiB-PET (**c**) values are expressed as standardized uptake value ratios (SUVRs). Regional measurements were extracted from BG regions of interest (ROIs) using automated atlas-based segmentations performed in each subject’s native imaging space and matched to the corresponding postmortem neuropathological hemisphere and anatomical region. Abbreviations: BG, basal ganglia; GPi, globus pallidus internus; GPe, globus pallidus externus; Pu, putamen; AD, Alzheimer’s disease; L-AD, low-likelihood Alzheimer’s disease; H-AD, high-likelihood Alzheimer’s disease; PSP, progressive supranuclear palsy; CBD, corticobasal degeneration; PiD, Pick’s disease; GGT, globular glial tauopathy; MD, mean diffusivity; SUVR, standardized uptake value ratio. PSP(1–4) represents the average values across all PSP cases, and GGT(1–2) represents the average values across all GGT cases. These pooled values are provided for descriptive visualization only and should not be interpreted as evidence of homogeneous group-level effects or as substitutes for the individual subject data presented elsewhere in the manuscript.

**Figure 10:**
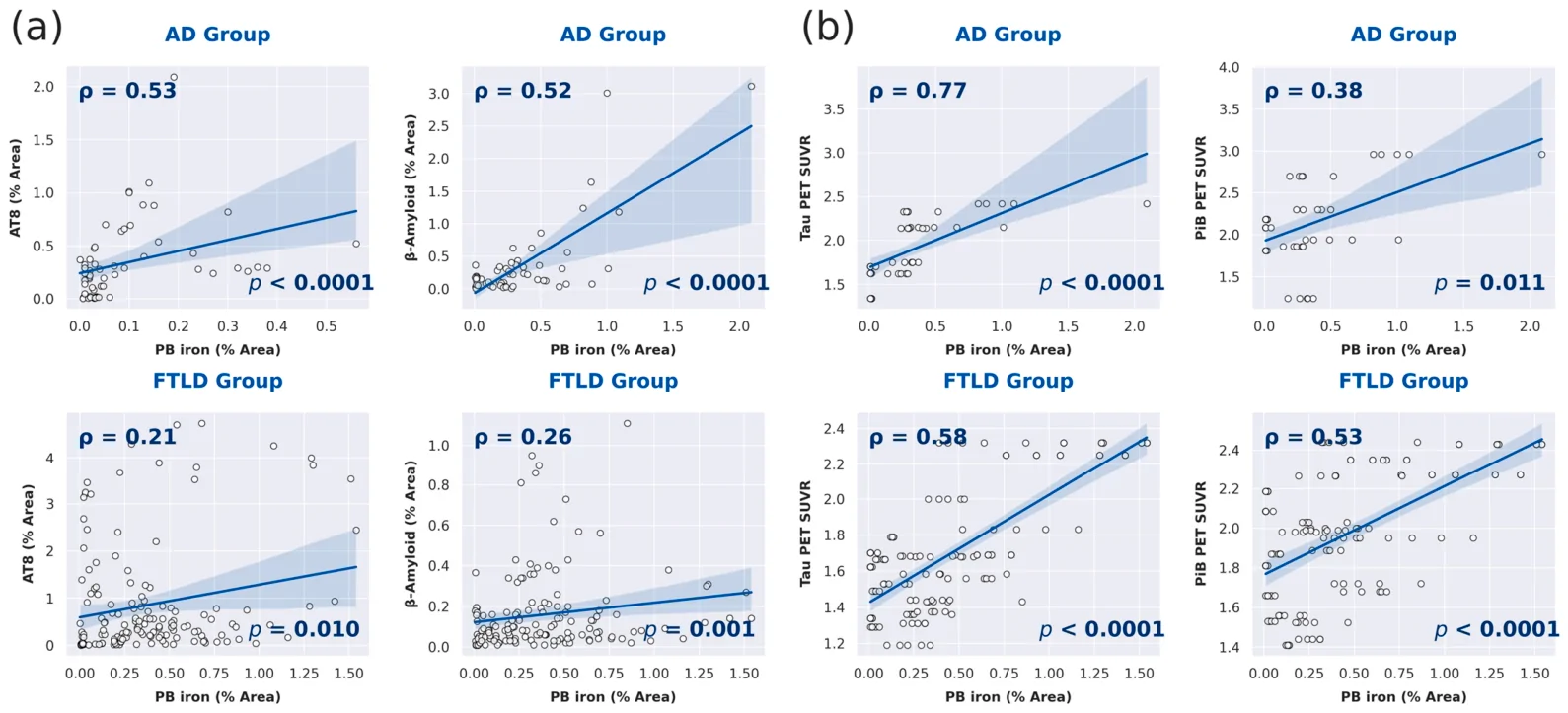
Correlative analysis between iron deposition, histopathological burden, and PET biomarkers in BG subregions. (**a**) Correlations between iron deposition, quantified by Prussian blue (PB) staining, and pathological burden measured by AT8 tau immunoreactivity and β-amyloid pathology across combined BG subregions, including the globus pallidus internus (GPi), globus pallidus externus (GPe), and putamen (Pu). Analyses are shown separately for the Alzheimer’s disease (AD) group (**upper** panels) and the frontotemporal lobar degeneration (FTLD) group (**lower** panels), with control cases included. (**b**) Correlations between PB staining and in vivo PET biomarkers, including tau PET and PiB-PET standardized uptake value ratios (SUVRs), across corresponding BG subregions. Results are presented separately for the AD group (**upper** panels) and the FTLD group (**lower** panels), with controls included in the analyses. Abbreviations: PB, Prussian blue; BG, basal ganglia; GPi, globus pallidus internus; GPe, globus pallidus externus; Pu, putamen; SUVR, standardized uptake value ratio; AD, Alzheimer’s disease; FTLD, frontotemporal lobar degeneration. Note: Correlation analyses were performed using regional measurements from 12 subjects. Each subject contributed measurements from up to three anatomically matched basal ganglia regions (GPi, GPe, and putamen), consistent with the study design described in Section 2.6. Therefore, the number of plotted observations exceeds the number of independent subjects, and multiple observations may originate from the same individual. Subject-level regional correlation coefficients are presented separately in Supplementary Figure S2.

**Table 1:** Patient demographics.

Neuropathological Diagnosis		Sex	Age at Onset	Age at PET	Age at Death	Interval PET to Death	Braak Stage	Thal Phase	ADNC	Vascular Score
1	Control-1	M	-	83	84	1	0	0	A0 B0 C0	2
2	Control-2	F	-	98	103	5	I	0	A0 B1 C0	1
3	Low-likelihood (L-AD)	F	63	77	81	4	I	III	A1 B1 C0	2
4	High-likelihood (H-AD)	M	58	75	78	3	VI	IV	A3 B3 C3	2
5	PSP-1 (PSP-RS)	M	64	66	68	2	I	0	A0 B1 C0	0
6	PSP-2 (PSP-SL)	M	72	82	83	1	II	0	A0 B1 C0	2
7	PSP-3 (PSP-SL)	F	80	86	86	1	IV	III	A2 B2 C1	2
8	PSP-4 (PSP-SL)	M	73	82	82	1	III–IV	III	A2 B2 C3	1
9	Corticobasal Degeneration (CBD)	F	73	73	75	2	I	0	A0 B1 C0	1
10	GGT-1 (Globular glial tauopathy—type I)	F	81	83	87	4	II	III	A2 B1 C1	4
11	GGT-2 (Globular glial tauopathy—type II)	M	52	57	61	4	0	0	A0 B0 C0	2
12	Pick’s body disease (PiD)	F	63	71	75	4	0	0	A0 B0 C0	2

Footnote. Description of patient demographics selected for the study. Abbreviations: M, male; F, female; L-AD, Low-likelihood Alzheimer’s disease; H-AD, High-likelihood Alzheimer’s disease; ADNC, Alzheimer’s Disease Neuropathological Change; PSP, progressive supranuclear palsy; CBD, Corticobasal Degeneration; PiD, Pick’s Disease; GGT, Globular glial tauopathy. Note: The vascular score accredited to each case consists of a five-point scale accounting for the presence or severity of four vascular lesion types: arteriolosclerosis, cerebral amyloid angiopathy (CAA), cortical microinfarcts, and lacunar or large infarcts. A score of 0 shows no vascular lesions present; 1 equals mild arteriolosclerosis or CAA only; 2 shows moderate–severe arteriolosclerosis or CAA only; 3 represents the presence of cortical microinfarcts without lacunar or large infarcts; and 4 indicates the presence of lacunar or large infarcts [62].

## Data Availability

Data are available from the corresponding author upon request due to privacy and ethical restrictions.

## Supplementary Materials

The following supporting information can be downloaded at: https://www.mdpi.com/article/10.3390/brainsci16101013/s1, Figure S1: Colocalization of Truncated 4R Tau (RD4) and Ferritin in Representative Basal Ganglia Subregions; Figure S2: Subject-Level Regional Spearman Correlations Between Prussian Blue Iron Burden and Neuropathological/PET Biomarkers Across Basal Ganglia Subregions; Table S1: Pathological and Colocalization Measures Stratified by Sex.
