# Supplementary material for: Pilot Multimodal Imaging Assessment of Neuropathological Iron Deposition and Molecular PET in Frontotemporal Lobar Degeneration
Source: Brain Sci. 2026 Sep 24;16(10):1013. doi: 10.3390/brainsci16101013 (PMC13702597; doi:10.3390/brainsci16101013)
Supplement: Supplementary file 1 [file brainsci-16-01013-s001.zip › brainsci-4537312-supplementary.pdf]

Figure S1. Colocalization of Truncated 4R Tau (RD4) and Ferritin in Representative Basal Ganglia Subregions

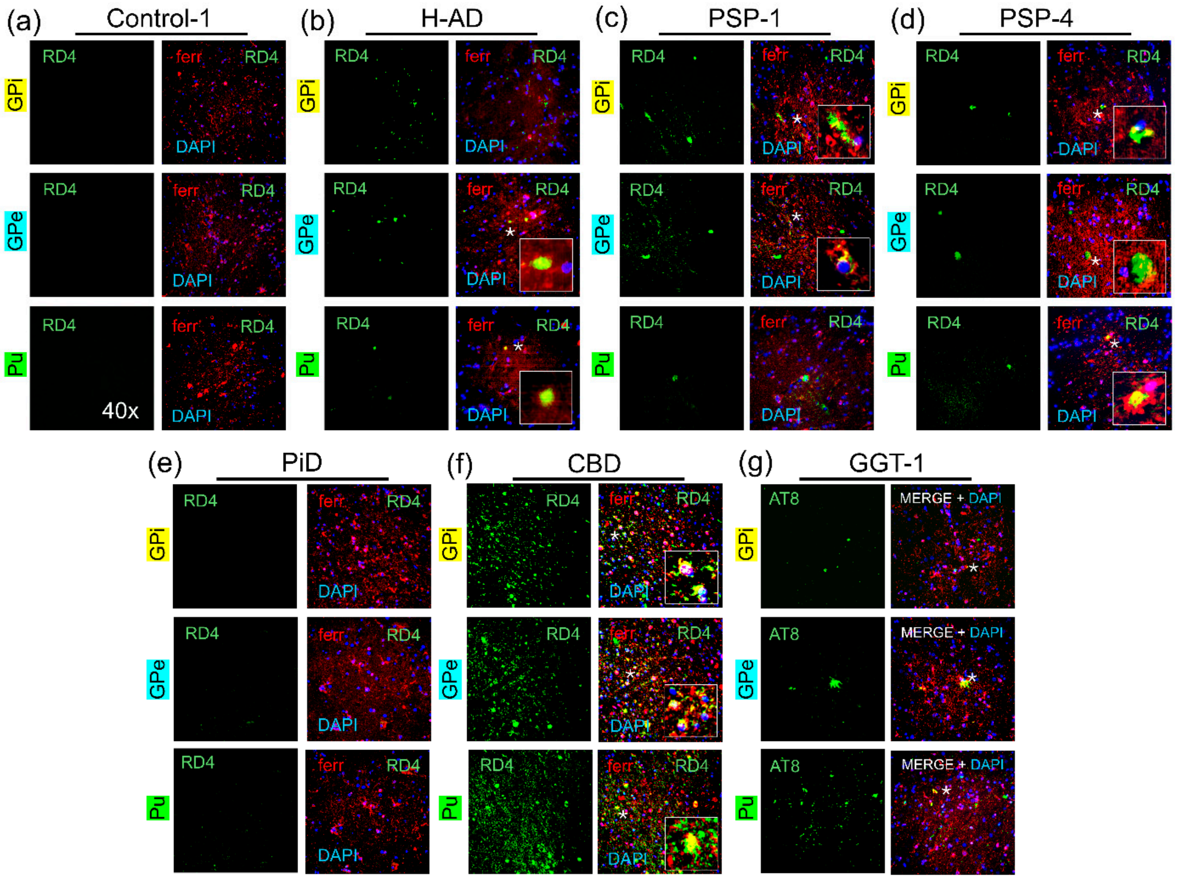

Footnote: Double immunofluorescence staining for truncated 4-repeat tau (RD4, green), ferritin (red), and DAPI (blue) in basal ganglia (BG) regions from control and tauopathy cases. Representative images from the globus pallidus internus (GPi), globus pallidus externus (GPe), and putamen (Pu) are shown for **(a)** Control-1, **(b)** high-likelihood Alzheimer's disease (H-AD), **(c)** PSP-1, (d) PSP-4, **(e)** Pick's disease (PiD), **(f)** corticobasal degeneration (CBD), and (g) GGT-1. RD4-positive tau pathology was sparse or absent in the control and PiD cases, whereas RD4 immunoreactivity was readily observed in PSP, CBD, and GGT cases. Merged images demonstrate the spatial relationship between RD4-positive tau pathology and ferritin immunoreactivity. Regions exhibiting RD4/ferritin colocalization (yellow signal) are indicated by white asterisks and shown at higher magnification in the corresponding inset panels. Colocalized RD4-positive inclusions and ferritin were observed across multiple BG regions in PSP, CBD, and GGT cases, with particularly abundant RD4 pathology in the CBD case. Nuclei were counterstained with DAPI. Images were acquired at 40× magnification. **Abbreviations:** GPi, globus pallidus internus; GPe, globus pallidus externus; Pu, putamen; PSP, progressive supranuclear palsy; PiD, Pick's disease; CBD, corticobasal degeneration; GGT, globular glial tauopathy.

Figure S2. Subject-Level Regional Spearman Correlations Between Prussian Blue Iron Burden and Neuropathological/PET Biomarkers Across Basal Ganglia Subregions

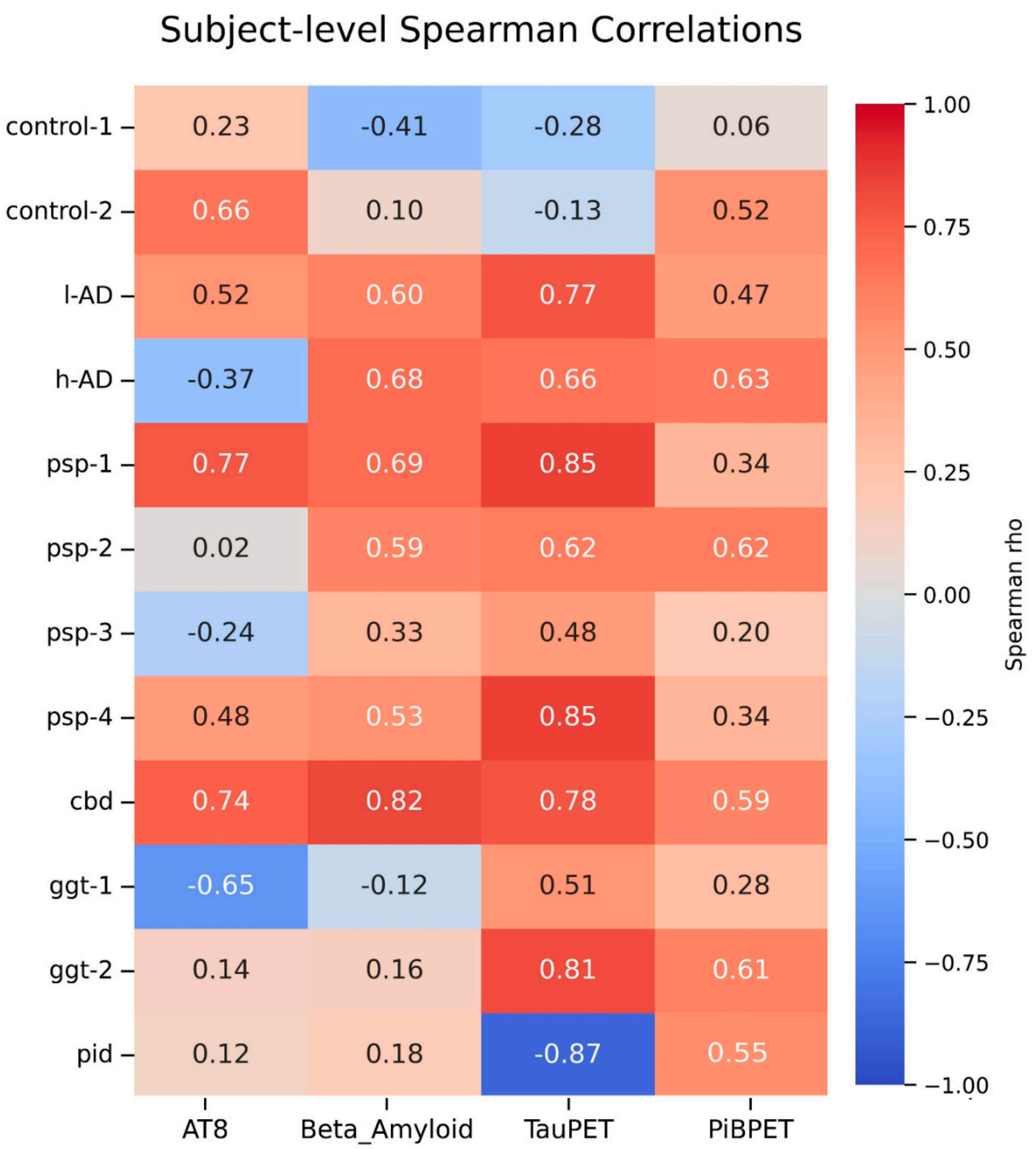

Footnote: Heatmaps display subject-level Spearman correlation coefficients (ρ) between regional Prussian blue (PB) staining and corresponding measures of AT8 tau pathology, β-amyloid burden, Tau-PET uptake, and PiB-PET uptake across the globus pallidus internus (GPi), globus pallidus externus (GPe), and putamen (Pu). Correlations are presented as exploratory effect-size estimates for individual cases to preserve biological heterogeneity and avoid reliance solely on pooled subgroup summaries. The data is consistent with a descriptive descriptive and hypothesis-generating purposes, as regional observations within subjects are not fully independent and the cohort is limited to 12 pathologically characterized cases. Findings should therefore be interpreted as associations rather than evidence of causal or disease-specific effects. **Abbreviations:** AT8, phosphorylated tau; Tau-PET, flortaucipir positron emission tomography; PiB-PET, Pittsburgh compound B positron emission tomography; GPi, globus pallidus internus; GPe, globus pallidus externus; Pu, putamen

Table S1. Pathological and Colocalization Measures Stratified by Sex

Variable	Male (n=6)	Female (n=6)
PB (%)	0.48	0.44
AT8 (%)	0.72	0.85
PCC	0.21	0.18

Footnote: Descriptive averages of regional Prussian blue (PB) iron burden, AT8-immunoreactive tau pathology, and Pearson's colocalization coefficients (PCC) between AT8 and ferritin stratified by biological sex. Overall, female subjects exhibited slightly higher AT8 pathology, whereas PB and PCC values were generally similar between sexes. Owing to the small sample size, unequal representation of pathological subtypes within each sex, incomplete colocalization data for some subjects, and the substantial clinicopathological heterogeneity of the cohort, formal statistical comparisons were not performed. Values are therefore presented for descriptive purposes only and should not be interpreted as evidence of sex-specific differences. **Abbreviations:** PB, Prussian blue; AT8, phosphorylated tau; PCC, Pearson's colocalization coefficient.
